# Histone methyltransferase Smyd2 drives vascular aging by its enhancer-dependent activity

**DOI:** 10.18632/aging.204449

**Published:** 2022-12-28

**Authors:** Zhenghua Su, Haibi Su, Jie Xu, Gang Wei, Lefeng Qu, Ting Ni, Di Yang, Yizhun Zhu

**Affiliations:** 1 School of Pharmacy, Pharmacophenomics Laboratory, Human Phenome Institute, Zhangjiang Fudan International Innovation Center, State Key Laboratory of Genetic Engineering, School of Life Sciences, Shanghai Key Laboratory of Bioactive Small Molecules, Fudan University, Shanghai 201203, P.R. China; 2 State Key Laboratory of Quality Research in Chinese Medicine and School of Pharmacy, Macau University of Science and Technology, Macau 999078, P.R. China; 3 Department of Vascular and Endovascular Surgery, Second Affiliated Hospital of Naval Medical University, Shanghai 200003, China

**Keywords:** vascular aging, Smyd2, H3K4me1, enhancer-dependent activity

## Abstract

Background: Vascular aging is one of the important factors contributing to the pathogenesis of cardiovascular diseases. However, the systematic epigenetic regulatory mechanisms during vascular aging are still unclear. Histone methyltransferase SET and MYND domain-containing protein 2 (Smyd2) is associated with multiple diseases including cancer and inflammatory diseases, but whether it is involved in endothelial cell senescence and aging-related cardiovascular diseases has not been directly proved. Thus, we aim to address the effects of Smyd2 on regulating angiotensin II (Ang II)-induced vascular endothelial cells (VECs) senescence and its epigenetic mechanism.

Methods and Results: The regulatory role of Smyd2 in Ang II-induced VECs senescence was confirmed by performing loss and gain function assays. Chromatin immunoprecipitation-sequencing (ChIP-seq) analysis was used to systematically screen the potential enhancer during VECs senescence. Here, we found that Smyd2 was significantly upregulated in Ang II-triggered VECs, and deficiency of Smyd2 attenuated senescence-associated phenotypes both *in vitro* and *in vivo*. Mechanically, Ang II-induced upregulation of Smyd2 could increase the mono-methylation level of histone 3 lysine 4 (H3K4me1), resulting in a hyper-methylated chromatin state, then further activating enhancers adjacent to key aging-related genes, such as *Cdkn1a* and *Cdkn2a*, finally driving the development of vascular aging.

Conclusions: Collectively, our study uncovered that Smyd2 drives a hyper-methylated chromatin state via H3K4me1 and actives the enhancer elements adjacent to key senescence genes such as *Cdkn1a* and *Cdkn2a*, and further induces the senescence-related phenotypes. Targeting Smyd2 possibly unveiled a novel therapeutic candidate for vascular aging-related diseases.

## INTRODUCTION

Aging accelerates the progress of vascular endothelial dysfunction, which is an independent risk factor for cardiovascular diseases [1]. Vascular endothelial cells (VECs) have been well documented to maintain vascular homeostasis and physiological function [2]. With age, senescent VECs accumulate and gradually develop into acute endothelial injury or proinflammatory state, which causes endothelial dysfunction and results in hypertension, atherosclerosis, vascular obstruction, and other vascular aging-related diseases [3, 4]. Clearance of senescent cells delays senescence-related phenotypes and extends the healthy lifespan [5]. However, the mechanisms underlying aging-induced endothelial dysfunction are not yet clarified. Thus, to develop new therapeutic interventions of age-related vascular lesions, understanding the factors that regulate VECs senescence is of paramount importance for the prognosis and diagnosis of human cardiovascular disorders.

VECs senescence undergoes morphological and functional changes including the flatter and enlarged cell morphology, the irreversible cell cycle arrest state, and chromatin alterations. Senescent VECs represent high levels of senescence-associated β galactosidase (SA-β-Gal) activity [6, 7], increased expressions of p53, p21^CIP1/WAF1^ and p16^INK4a^ as well as proinflammatory cytokines, which were known as the chronic senescence-associated secretory phenotypes (SASP) [8]. Despite the contributing role of VECs senescence in the development of cardiovascular diseases, global studies of chromatin structure and function in vascular endothelium have to date not been undertaken.

Over the past few years, interest in epigenetics has surfaced with accelerated emphasis. Compared to normal cells, senescent cells undergo dramatic epigenetic alterations, such as histone modifications, microRNAs, and chromatin remodeling, which further modulate vascular aging-related phenotypes [9, 10]. Histone methyltransferase Smyd2 belongs to the SMYD family [11] and has been recognized to mono-methylate histone 3 lysine 4 (H3K4me1) and di-methylate histone 3 lysine 36 (H3K36me2) [12]. Furthermore, Smyd2 also methylates non-histone proteins, such as the tumor-suppresser proteins p53 and RB1, etc. [13, 14]. Recently, Smyd2-mediated TRAF2 or Sphk/S1PR methylation aggravate inflammatory responses or ischemic stroke, respectively [15, 16]. Moreover, the methylation status of the Smyd2 promoter is also associated with the progress of abdominal aortic aneurysm (AAA), whose morbidity increases with advancing age in men [17]. However, the effect of Smyd2 on vascular endothelial senescence have not to our knowledge been explored.

Enhancers are defined as the cis-regulatory elements situated at distal regulatory regions that effectively drive gene transcription through specific chromatin conformations [18]. Enhancers are activated by the relaxation of compact chromatin and dynamically regulated by transcription factors (TFs) binding or enhancer-specific histone modifications [19], such as H3K27ac which marks active enhancers, and H3K4me1 which marks inactive, active, or poised enhancers [20]. Researches recently reveal that senescence-activated enhancers positively regulate SASP expressions and accelerate mouse embryonic fibroblasts (MEFs) replicative senescence [21]. Moreover, the vascular enhancer repertoires are also altered in both Ang II-induced VSMCs and Ang II-infused mice [22]. However, the relationship between epigenetic alteration and enhancer activation in Ang II-triggered VECs senescence remains uncharacterized.

In the present study, we focused on Smyd2, which can mono-methylate histone 3 lysine 4 (H3K4me1), was profoundly upregulated in Ang II-induced VECs, and Smyd2 ablation attenuated senescence-associated phenotypes both *in vitro* and *in vivo*. Mechanically, Ang II-induced upregulation of Smyd2 activated H3K4me1 marks and further promoted endothelial senescence via activating enhancers adjacent to key aging-related genes, such as *Cdkn1a* (encoding p21 protein) and *Cdkn2a* (encoding p16 protein). Taken together, targeting Smyd2 has important implications in epigenetically treating VECs senescence and vascular aging-associated diseases.

## RESULTS

### Smyd2 is upregulated in vascular aging both *in vivo* and *in vitro*

**Figure 1:**
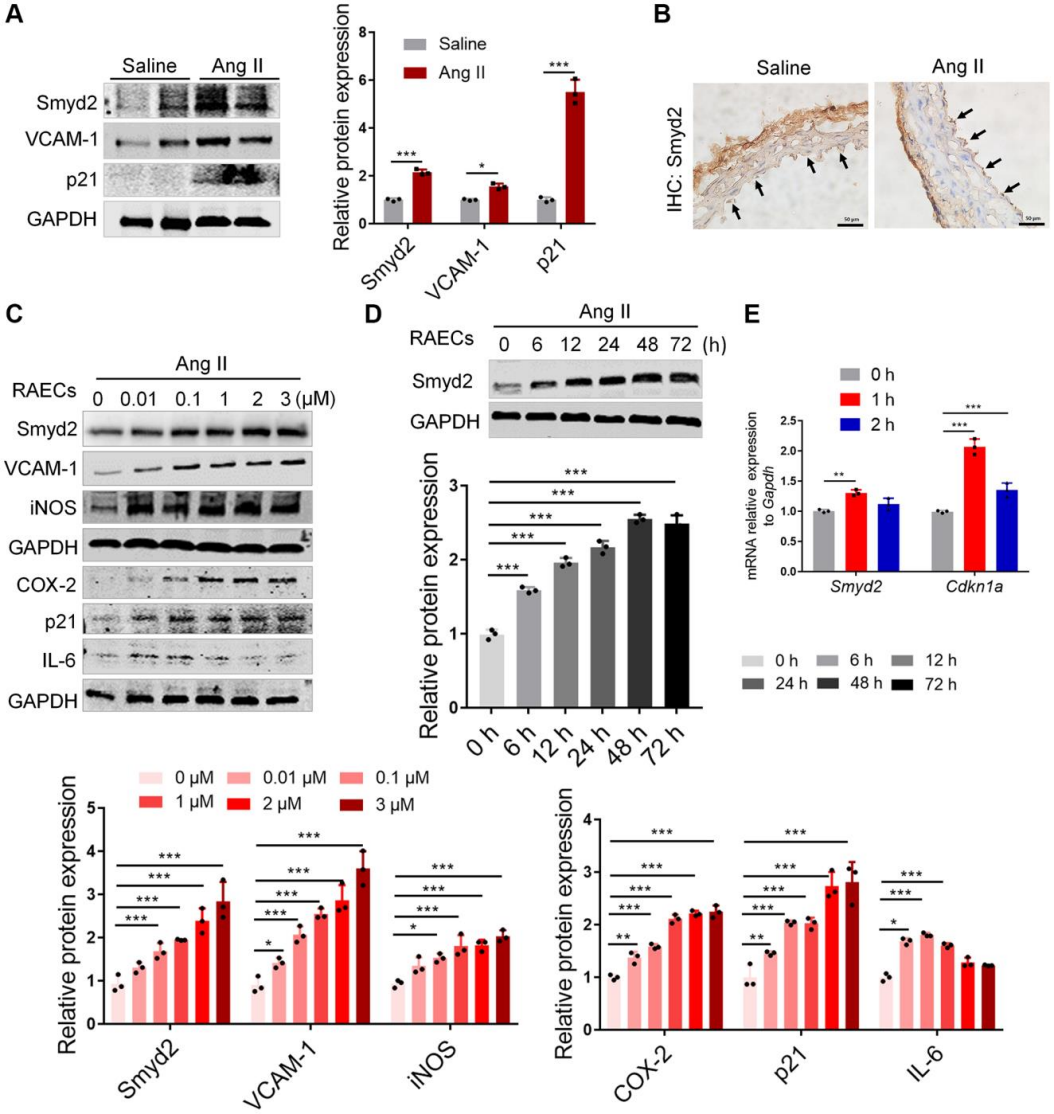
**Smyd2 is upregulated in vascular aging both *in vivo* and *in vitro*.** (**A**, **B**) The *in vivo* aging model was established by Ang II infusion in mice for 28 days and harvested the aortas. The protein expressions of Smyd2, VCAM-1, and p21 were detected by immunoblots (**A**). The immunohistochemistry assay was performed with Smyd2 antibody. Scale bars, 50 μm (**B**). (**C**–**E**) Ang II (100 nM, 48 h)-induced RAECs was used as the *in vitro* aging model. Smyd2 and the senescence-related phenotype markers were increased in a dose-dependent manner in Ang II-induced RAECs (**C**). The protein expression of Smyd2 was increased in a time-dependent manner in Ang II-induced RAECs (**D**). RT-qPCR analysis of *Smyd2* and *Cdkn1a* genes in Ang II-induced RAECs (**E**). Data are presented as the mean ± SEMs, ^*^p < 0.05, ^**^p < 0.01, ^***^p < 0.001, each acquired from three individual experiments (*n* = 3).

To dissect the relationship between Smyd2 and vascular aging, we established the *in vivo* aging model by infusion of saline or Ang II in mice for 28 days as reported in our previous study [23]. Evidence of aortic aging in Ang II-infused mice was confirmed by upregulation of p21 and VCAM-1 protein levels (Figure 1A). Surprisingly, the expression of Smyd2 increased in response to Ang II infusion as detected by both immunoblots and immunohistochemistry assays (Figure 1A, 1B).

To further investigate the role of Smyd2 in VECs senescence, we stimulated both rat primary aorta endothelial cells (RAECs) [23] and human umbilical vein endothelial cells (HUVECs) with Ang II for the indicated dose and time. As expected, Ang II led to Smyd2 upregulation in both time- and dose-dependent manner, accompanied by a progressive increase of senescence-related phenotypes in both RAECs (Figure 1C, 1D) and HUVECs (Supplementary Figure 1A, 1B). In addition, the mRNA level of *Smyd2* and *Cdkn1a* (encoding p21 protein) genes were also increased in Ang II-induced RAECs (Figure 1E). These above data suggest that upregulation of Smyd2 was associated with Ang II-induced vascular aging both *in vivo* and *in vitro*.

### Knockdown of Smyd2 attenuates Ang II-induced RAECs senescence

**Figure 2:**
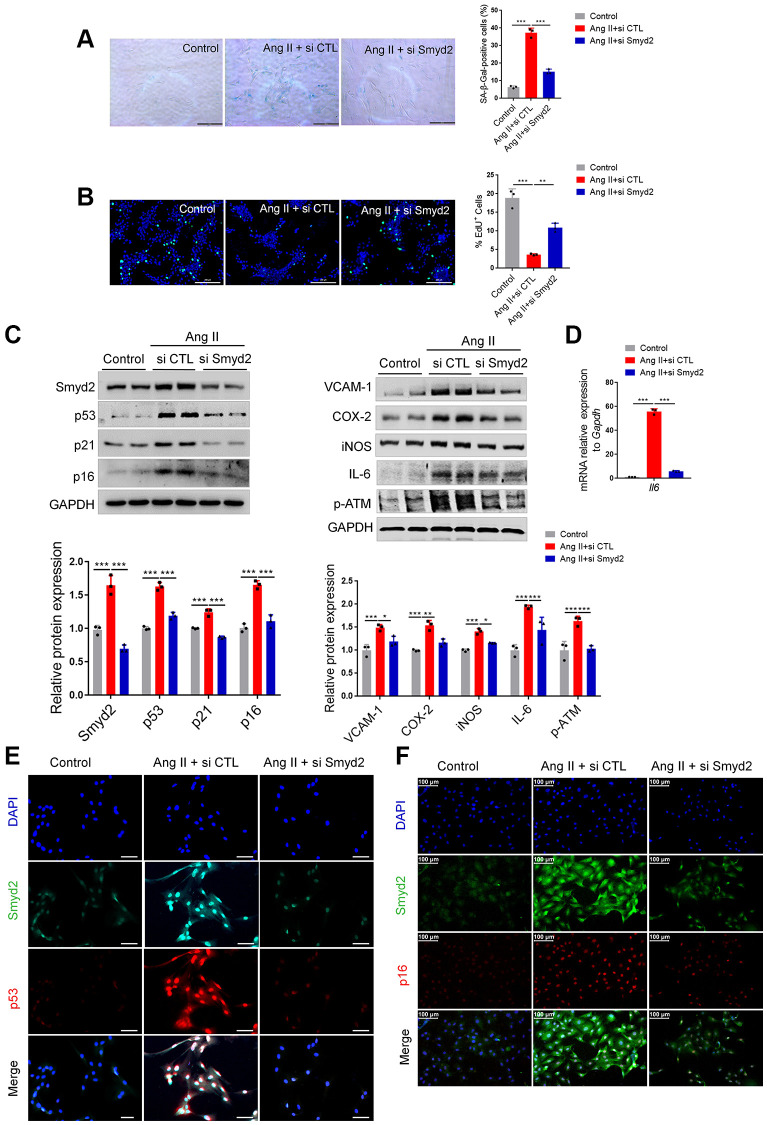
**Knockdown of Smyd2 attenuates Ang II-induced RAECs senescence.** Smyd2 was knocked down using si Smyd2 in Ang II-induced RAECs, si CTL was used as the negative control. (**A**, **B**) The SA-β-gal staining and EdU incorporation assay. Representative staining images are shown in the figure left, and the statistical analysis of positive cells is shown on the right. Scale bars, 200 μm. (**C**) Western blot analysis of Smyd2 and the senescence markers (p53, p21 and p16), the proinflammatory mediators (iNOS, VCAM-1, COX-2 and IL-6) and the DNA damage marker (p-ATM) protein. Representative western blot images are shown in the figure above, and the statistical analysis of relative protein expression is shown below. GAPDH was used as the loading control. (**D**) The mRNA expression of *Il6* gene by RT-qPCR analysis. (**E**, **F**) Immunofluorescence double staining of Smyd2 and the senescence markers (p53 and p16). Scale bars, 100 μm. Data are presented as the mean ± SEMs, ^*^p < 0.05, ^**^p < 0.01, ^***^p < 0.001, each acquired from three individual experiments (*n* = 3).

To explore the regulatory role of Smyd2 in vascular endothelial senescence, we knocked down Smyd2 in RAECs using small interfering RNA (siRNA) (si Smyd2). Interestingly, a substantial increase in SA-β-Gal activity and a decrease in EdU incorporation, the markers of senescent cells, were observed in Ang II-induced RAECs, which were significantly reversed after Smyd2 siRNA treatment (Figure 2A, 2B). Moreover, Smyd2 knockdown also dramatically downregulated the senescence-associated phenotypes, including the senescence markers (p53, p21, and p16), the proinflammatory mediators (iNOS, VCAM-1, COX-2, and IL-6) and the DNA damage marker (p-ATM) (Figure 2C). As well, the increased mRNA level of the *Il6* gene (encoding IL-6 protein) in Ang II-induced RAECs was abrogated by Smyd2 knockdown (Figure 2D). Furthermore, double immunofluorescence staining results showed the higher co-expressions of Smyd2 with p53 or p16 in Ang II-induced RAECs but attenuated by Smyd2 deficiency (Figure 2E, 2F). These results suggest that Smyd2 knockdown attenuates Ang II-induced RAECs senescence.

### Inhibition of Smyd2 alleviates Ang II-induced senescence-associated phenotypes *in vitro*

**Figure 3:**
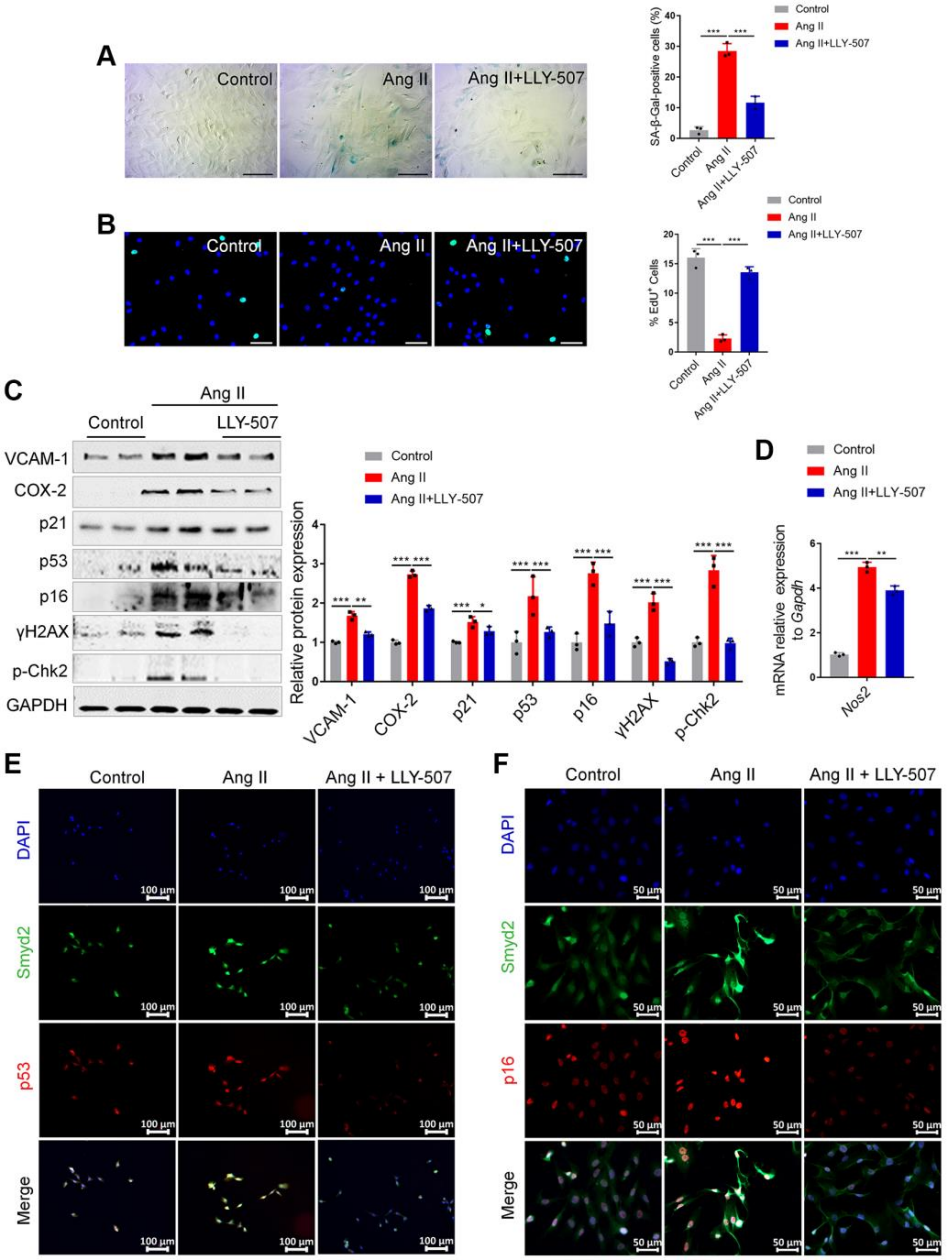
**Inhibition of Smyd2 alleviates Ang II-induced senescence associated phenotypes *in vitro*.** (**A**, **B**) SA-β-gal staining (**A**) and EdU incorporation (**B**) assays in Ang II-induced RAECs with LLY-507 (3 μM) pretreatment. Representative staining images are shown in the figure left, and the statistical analysis of positive cells is shown in the figure right. Scale bars, 100 μm. (**C**) Expressions of senescence markers (p53, p21 and p16), pro-inflammatory molecules (VCAM-1 and COX-2) and DNA damage markers (p-Chk2 and γH2AX) were detected by western blot analysis upon LLY-507 pretreatment in Ang II-induced RAECs. Representative western blot images are shown on the left, and the statistical analysis of relative protein expressions is shown on the right. GAPDH was used as the loading control. (**D**) The mRNA levels of *Nos2* (encoding the iNOS protein) in Ang II-induced RAECs with LLY-507 pretreatment was examined by RT-qPCR. (**E**, **F**) Immunofluorescence double staining of Smyd2 and the senescence markers (p53 and p16) upon LLY-507 pretreatment in Ang II-induced RAECs. Scale bars, 100 μm or 50 μm. Data are presented as the mean ± SEMs, ^*^p < 0.05, ^**^p < 0.01, ^***^p < 0.001, each acquired from three individual experiments (*n* = 3).

It is well documented that LLY-507 is a potent and selective inhibitor of Smyd2 and potently inhibits the ability of Smyd2 to methylate the p53 peptide [24]. Therefore, we evaluated whether LLY-507 could alleviate Ang II-induced RAECs senescence. It is noteworthy that decreased SA-β-Gal activity and increased EdU incorporation were observed upon LLY-507 treatment in Ang II-induced RAECs (Figure 3A, 3B). Furthermore, LLY-507 also reduced numerous senescence markers including the senescence markers (p53, p21, p16), the pro-inflammatory molecules (VCAM-1, COX-2), and the DNA damage markers (p-Chk2, γH2AX) (Figure 3C). In addition, the mRNA level of the *Nos2* gene (encoding the iNOS protein) was also ameliorated by LLY-507 (Figure 3D). Moreover, the immunofluorescence double-staining analysis results revealed that co-expressions of Smyd2 with p53 or p16 in Ang II-induced RAECs were markedly abrogated by treatment with LLY-507 (Figure 3E, 3F). All these above data suggest that LLY-507, a Smyd2 specific small molecule inhibitor, alleviates Ang II-induced senescence phenotypes *in vitro*.

### Overexpression of Smyd2 alone promotes the vascular endothelial senescence

**Figure 4:**
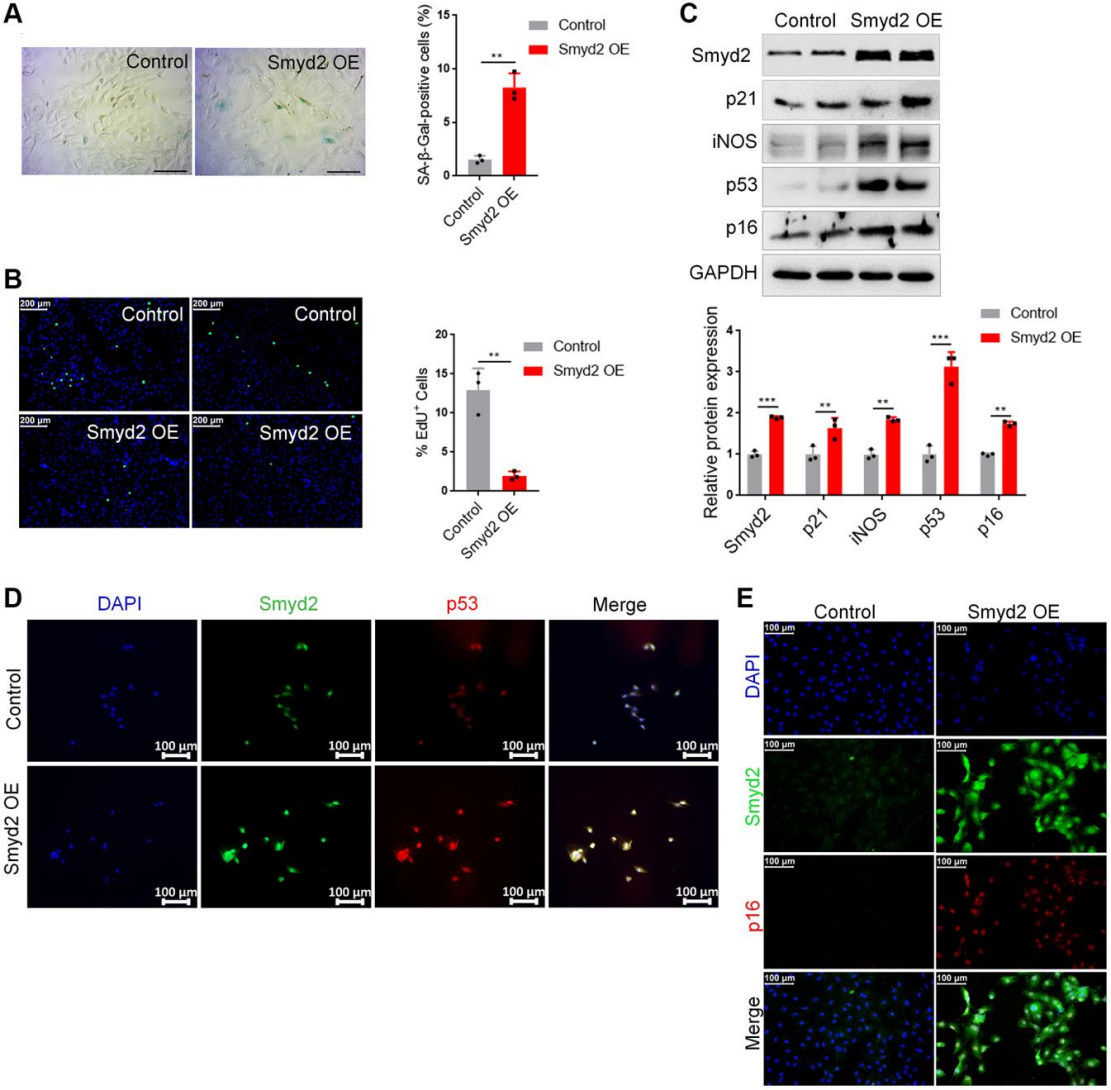
**Overexpression of Smyd2 alone promotes the vascular endothelial senescence.** Smyd2 was overexpressed (Smyd2 OE) in RAECs by transfecting with lentiviruses. (**A**, **B**) SA-β-gal staining (**A**) and EdU incorporation (**B**) assays in RAECs. Representative staining images are shown in the figure left, and the statistical analysis of positive cells is shown in the figure right. (**C**) Immunoblots of the senescence-associated markers (p53, p21, p16 and iNOS) proteins were detected by western blot assay, and the statistical analysis of relative protein expression is shown below. GAPDH was used as the loading control. (**D**, **E**) Immunofluorescence double staining of Smyd2 and p53 or p16 protein in RAECs transduced with lentiviruses expressing Smyd2. Scale bars, 100 μm. Data are presented as the mean ± SEMs, ^*^p < 0.05, ^**^p < 0.01, ^***^p < 0.001, each acquired from three individual experiments (*n* = 3).

To explore whether the increased Smyd2 could directly result in vascular aging, we overexpressed Smyd2 by transfecting Smyd2 overexpression lentiviruses (Smyd2 OE) in RAECs. Smyd2 OE led to apparent senescence-related phenotypes, such as an increased percentage of positive SA-β-Gal staining cells (Figure 4A) and decreased EdU incorporation (Figure 4B). Meanwhile, the senescence markers such as p53, p21, p16, and iNOS were also augmented after Smyd2 OE treatment (Figure 4C). The immunofluorescence analysis assays with Smyd2 and p53 or p16 antibodies also provided corroborating evidence (Figure 4D, 4E), which suggest that Smyd2 alone directly triggered the vascular endothelial senescence.

### Smyd2 knockdown or heterozygous knockout mice ameliorate senescence-associated phenotypes upon Ang II infusion

To further determine the effects of increased Smyd2 during Ang II-infused aging mice, we performed lentivirus-mediated knockdown of Smyd2 (sh*Smyd2*) in Ang II-infused mice. The results showed that the expression of Smyd2 was decreased in artery sections of sh*Smyd2*-treated mice versus shMock-treated mice after Ang II infusion, which was also accompanied by reduced expressions of the senescence marker (p53) and the pro-inflammatory molecules (iNOS and VCAM-1) (Supplementary Figure 2A).

**Figure 5:**
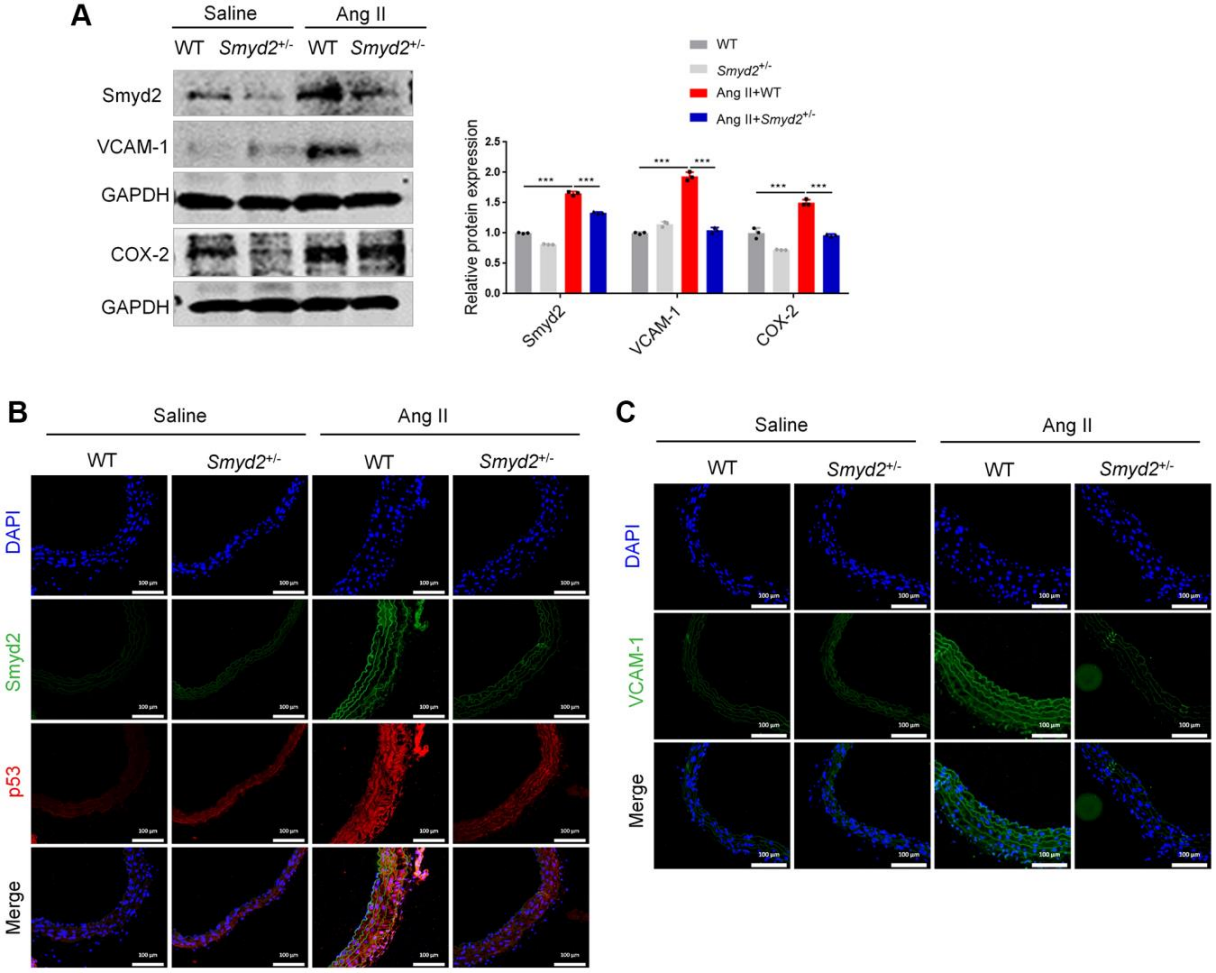
**Smyd2 heterozygous knockout mice ameliorates senescence-associated phenotypes upon Ang II infusion.*** Smyd2*^+/−^ mice and their wild-type littermates (WT) were infused with Ang II for 4 weeks. (**A**) Immunoblots of the proinflammatory mediators (COX-2 and VCAM-1). (**B**, **C**) The immunofluorescence analysis of Smyd2 with p53 or VCAM-1 in the cross-sectional area of blood vessels. Scale bars, 100 μm. Data are presented as the mean ± SEMs, ^***^p < 0.001, each acquired from three individual experiments (*n* = 3).

Taking it a step further, we constructed the Smyd2 heterozygous (*Smyd2*^+/−^) mice as described previously [15]. Western blot results verified the deficiency of Smyd2 in aorta tissues from *Smyd2*^+/−^ mice (Supplementary Figure 2B). Then *Smyd2*^+/−^ mice and the wild-type littermates (WT) were infused with saline or Ang II for 4 weeks. We found that the expressions of proinflammatory mediators (COX-2 and VCAM-1) were significantly increased in Ang II-infused WT mice, whereas this response was attenuated in *Smyd2*^+/−^ mice (Figure 5A). Moreover, the immunofluorescence analysis results revealed that co-expression of Smyd2 and p53 or VCAM-1 in the cross-sectional area of blood vessels were markedly blunted in *Smyd2*^+/−^ mice upon Ang II infusion compared with WT mice (Figure 5B, 5C). These findings described above strongly support that Smyd2 knockdown or heterozygous knockout mice ameliorate senescence-associated phenotypes upon Ang II infusion.

### Smyd2 upregulation activates distant enhancers adjacent to *Cdkn2a* (p16) and *Cdkn1a* (p21) genes

**Figure 6:**
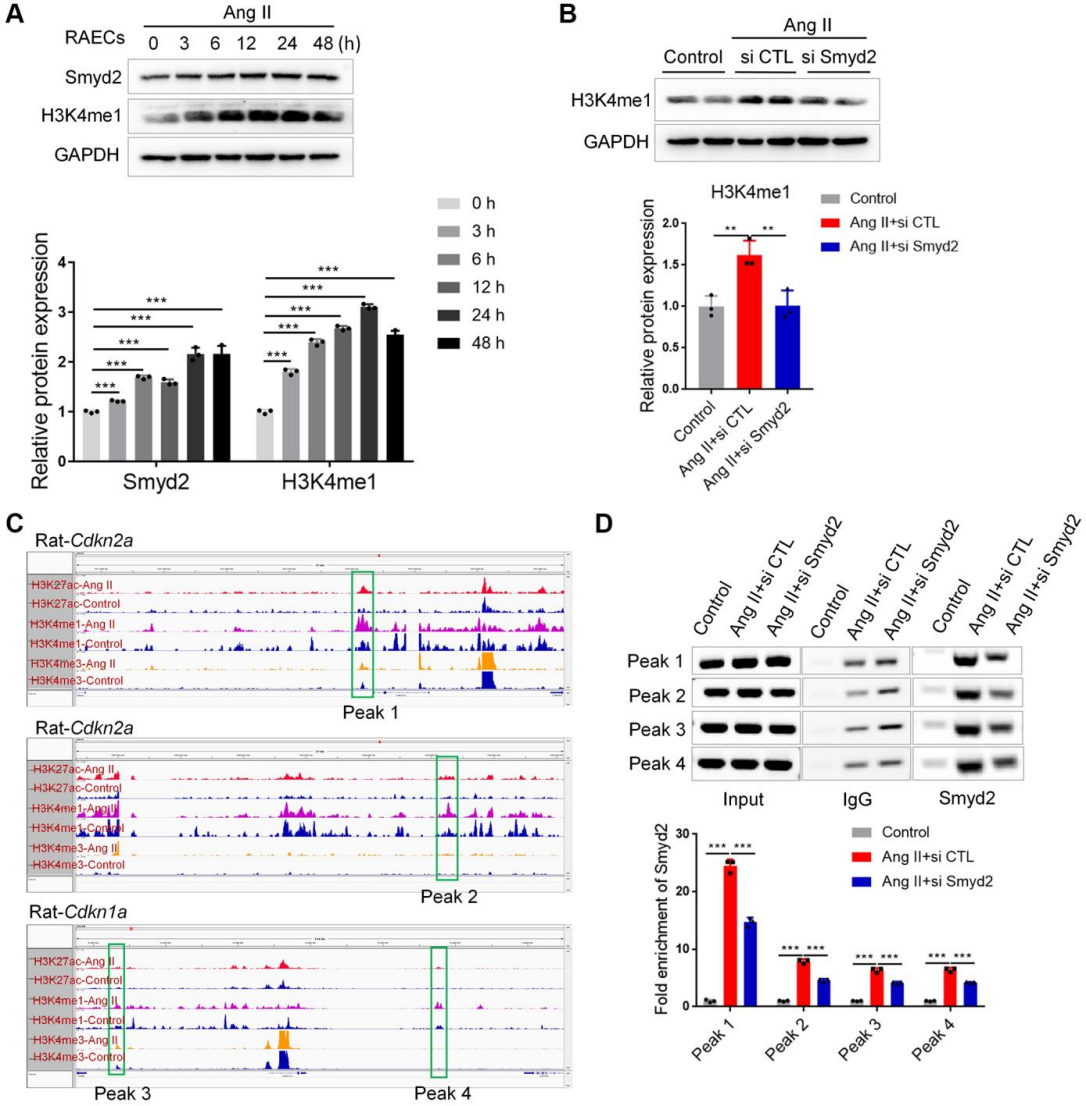
**Smyd2 upregulation activated distant enhancers adjacent to *Cdkn2a* (p16) and *Cdkn1a* (p21) genes.** (**A**) Western blot analysis of the Smyd2 and H3K4me1 level in Ang II-treated RAECs by the time-dependent manner. (**B**) The H3K4me1 level was detected after Smyd2 knockdown in Ang II-treated RAECs. (**C**) The ChIP-seq visualization data with H3K27Ac, H3K4me1 as well as H3K4me3 antibodies of *Cdkn2a* (p16) and *Cdkn1a* (p21) genes in Ang II-treated or control RAECs, as shown in Integrative Genomics Viewer (IGV). (**D**) The potential enhancer regions of *Cdkn2a* and *Cdkn1a* genes, namely peak 1–4, were evaluated by ChIP-PCR in Ang II-treated RAECs after Smyd2 knockdown. Data are presented as the mean ± SEMs, ^**^p < 0.01, ^***^p < 0.001, each acquired from three individual experiments (*n* = 3).

Next, we investigated the potential mechanism of Smyd2 regulating vascular senescence. Given that Smyd2 mono-methylates histone 3 lysine 4 (H3K4me1) to drive genes transcription and H3K4me1 marks inactive, active, and poised enhancers, we hypothesized that Ang II-triggered Smyd2 upregulation induced H3K4me1 enrichments and further activated distant enhancers adjacent to senescence-related genes. To verify this hypothesis, we examined the H3K4me1 level in Ang II-induced RAECs and found the level of H3K4me1 was upregulated upon Ang II treatment in a time-dependent manner (Figure 6A). Furthermore, the upregulated H3K4me1 could be abrogated by Smyd2 knockdown (Figure 6B), suggesting that Smyd2 regulated the level of H3K4me1 in Ang II-induced RAECs senescence.

To further characterize whether Ang II-upregulated H3K4me1 contributed to the activation of distant enhancers, we performed genome-wide chromatin immunoprecipitation coupled with deep sequencing (ChIP-seq) analysis following lysis of cross-linked RAECs upon Ang II treatment, using antibodies recognizing H3K27ac for active enhancers, H3K4me1 for candidate enhancers and H3K4me3 for active promoter regions to profile the global enhancer activities and investigate the molecular evidence of altered enhancers in Ang II-triggered RAECs. MACS2 was used to call peaks significantly enriched with ChIP-seq reads, using the *Q* value (corrected *p*-value) < 0.001 as the cutoff. Through the genome-wide chromatin profiling, we searched for active enhancers by identifying high H3K27ac/H3K4me1 co-occupied regions but low ChIP-seq signal of H3K4me3 in Ang II-induced RAECs. To eliminate the disturbance from the transcription starting site (TSS) or promoters, H3K4me1 and H3K27ac peaks should be at least 1 kb from a known TSS site or H3K4me3 peaks. Here we chose the peaks with changes of normalized coverage greater than 2-fold as changed peaks corresponding to Ang II treatment and then searched genome-wide for regions where H3K27ac and H3K4me1 had common upregulated peaks after Ang II treatment which represented the bona fide functional enhancers [25]. Upon the data analysis, we identified significantly increased H3K4me1 and H3K27ac enrichments upon Ang II treatment (a total of 11202 H3K27Ac, 48332 H3K4me1 as well as 13171 H3K4me3 detectable peaks in Ang II-induced senescent RAECs) (Supplementary Figure 3), indicating a possible activation of certain enhancers.

Then we searched for the appearance of enhancers adjacent to key senescence genes, such as *Cdkn1a* (p21) and *Cdkn2a* (p16). As shown in the ChIP-seq visualization data (Figure 6C), at least 4 remote enhancer elements engaged by Ang II were observed to locate at approximately 10 kb distal to *Cdkn2a* (p16) and *Cdkn1a* (p21) genes, which denoted as peaks 1–4. These peaks were at an active enhancer state which is characterized by high H3K27ac and H3K4me1 levels after Ang II treatment. Then we tested whether Smyd2 was recruited to these peaks and enhanced the H3K4me1 levels in Ang II-induced RAECs senescence. Indeed, ChIP-PCR data showed an increased Smyd2 binding after treatment with Ang II, but especially reversed by knockdown Smyd2 (si Smyd2) (Figure 6D). Overall, our results revealed a previously unrecognized capacity for Ang II to recruit Smyd2 to preexisting and de novo candidate enhancer elements distal to *Cdkn2a* (p16) and *Cdkn1a* (p21) genes, in a manner that partially depended on Smyd2 expression.

### Ang II-induced Smyd2 upregulation is mediated by the PI3K/Akt pathway

Studies have shown that inactivation of PI3K/Akt pathway is closely involved in the occurrence of vascular aging [26]. To explore the regulatory mechanism of increased Smyd2 expression in Ang II-induced RAECs senescence, the PI3K/Akt signaling pathway inhibitor LY294002 was used to block the PI3K/Akt pathway and detect the expression changes of Smyd2. As seen in Supplementary Figure 4, blocking the PI3K/Akt pathway with LY294002 significantly downregulated the protein level of Smyd2 in Ang II-induced RAECs, accompanied by decreased expressions of vascular senescence-related proteins, such as VCAM-1, iNOS, COX-2, and p16, demonstrating that Ang II-induced upregulation of Smyd2 may depend on the PI3K/Akt pathway.

### Smyd2 is associated with other vascular aging-related diseases

**Figure 7:**
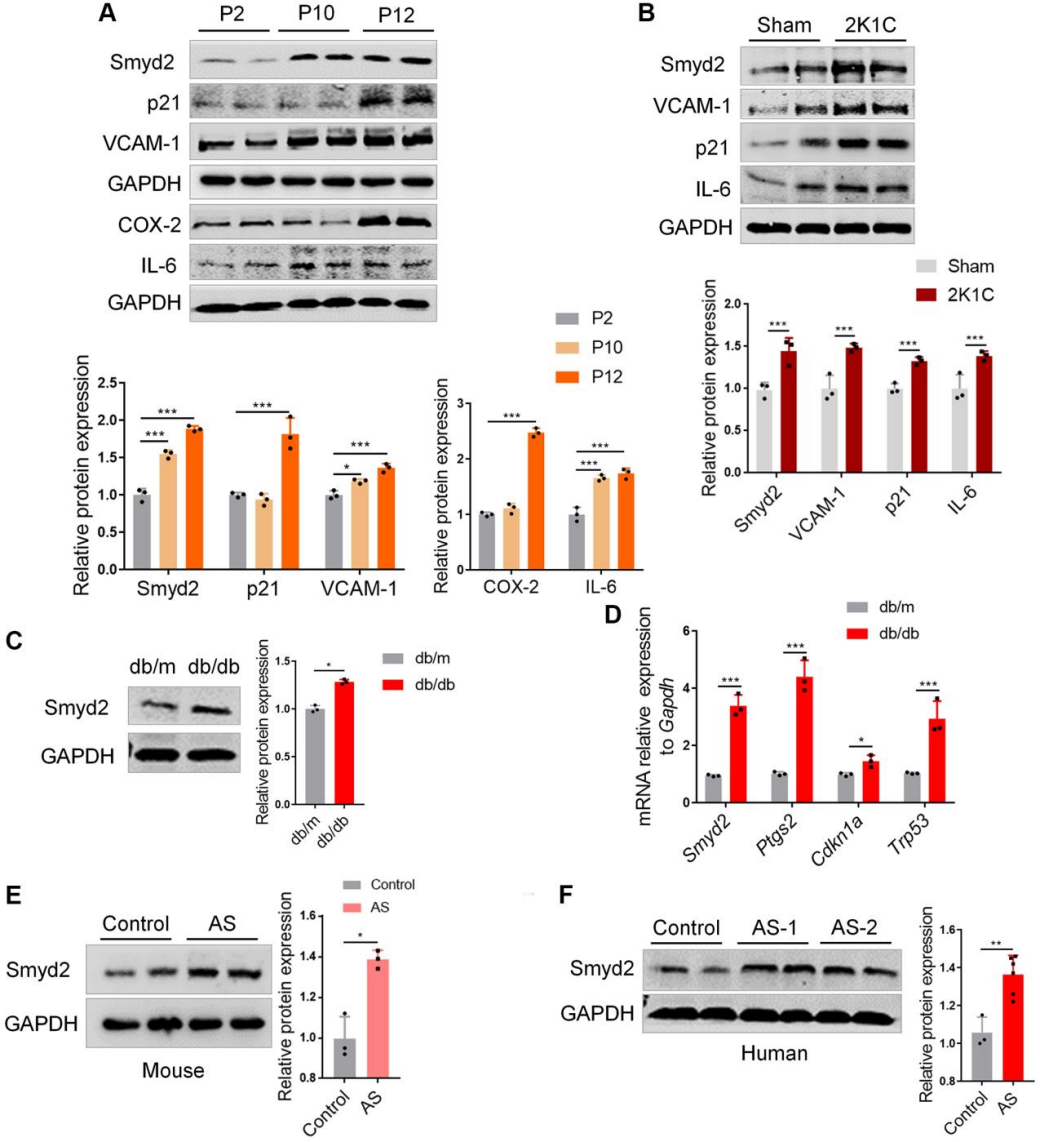
**Smyd2 is associated with other vascular aging-related diseases.** (**A**) The protein expressions of Smyd2, the senescence marker (p21) and the proinflammatory mediators (VCAM-1, COX-2 and IL-6) were examined in a later passage of RAECs by Western blot. GAPDH was used as the loading control. (**B**) Western blot analysis of Smyd2 and the senescence markers (p21, VCAM-1 and IL-6) in arteries of two-kidney one-clip renovascular hypertensive (2K1C) mice. The statistical analysis of relative protein expression is shown in the figure below. (**C**) Western blot analysis of Smyd2 in arteries of db/db mice and its control db/m mice. (**D**) The mRNA levels of Smyd2 and the senescence marker genes (*Ptgs2, Cdkn1a* and *Trp53*) in arteries of db/db mice and its control db/m mice. (**E**) The expression of Smyd2 in mice atherosclerotic artery samples was evaluated by western blot. (**F**) The protein level of Smyd2 in thoracic aorta in healthy (control) and atherosclerotic (AS) patients evaluated by western blot. Data are presented as the mean ± SEMs, *n* = 3 or 6. ^*^p < 0.05, ^**^p < 0.01, ^***^p < 0.001.

Previous studies revealed that the aged vascular endothelium was led by accumulated senescent VECs because of replicative exhaustion. This replicative senescence could be mimicked by the repeated passage of VECs *in vitro* [27]. Interestingly, here we found that the protein expression of Smyd2 profoundly increased at a later passage of RAECs and concomitant upregulation in the senescence marker (p21) and the proinflammatory mediators (VCAM-1, COX-2 and IL-6) (Figure 7A).

Emerging evidence has revealed that vascular endothelial senescence is a strong predictor of mortality in diverse vascular aging-related diseases such as atherosclerosis, hypertension, and diabetes [28], thus we are curious whether Smyd2 is associated with these vascular aging-related diseases. For hypertension, we constructed a two-kidney and one-clip renovascular hypertensive (2K1C) mice model [29] and found the expression of Smyd2 increased accompanied by upregulation of the senescence markers (p21, VCAM-1, and IL-6) (Figure 7B). For diabetes, the db/db mice and their control db/m mice were obtained and found the Smyd2 level was significantly enhanced in arteries of db/db mice compared with the db/m mice (Figure 7C). Moreover, RT-qPCR results also showed upregulation of *Smyd2* and the senescence marker genes, including *Ptgs2* (encoding COX-2 protein), *Cdkn1a* (encoding p21 protein) and *Trp53* (encoding p53 protein) (Figure 7D). For atherosclerosis, a western diet was used to feed the ApoE^−/−^ mice for 12 weeks to establish the classical atherosclerosis mouse model as we previously reported [30] and the entire aortas were harvested for analysis. We found a higher expression of Smyd2 in the aorta of atherosclerosis mice compared to that of control mice (Figure 7E). Besides, similar patterns were observed in the human atherosclerotic samples compared to the healthy thoracic aorta (Figure 7F). Taken together, upregulation of Smyd2 expression in vessel tissue is positively correlated with vascular aging in both humans and mice, indicating that Smyd2 may have an important impact on the progression of vascular aging-related diseases.

## DISCUSSION

**Figure 8:**
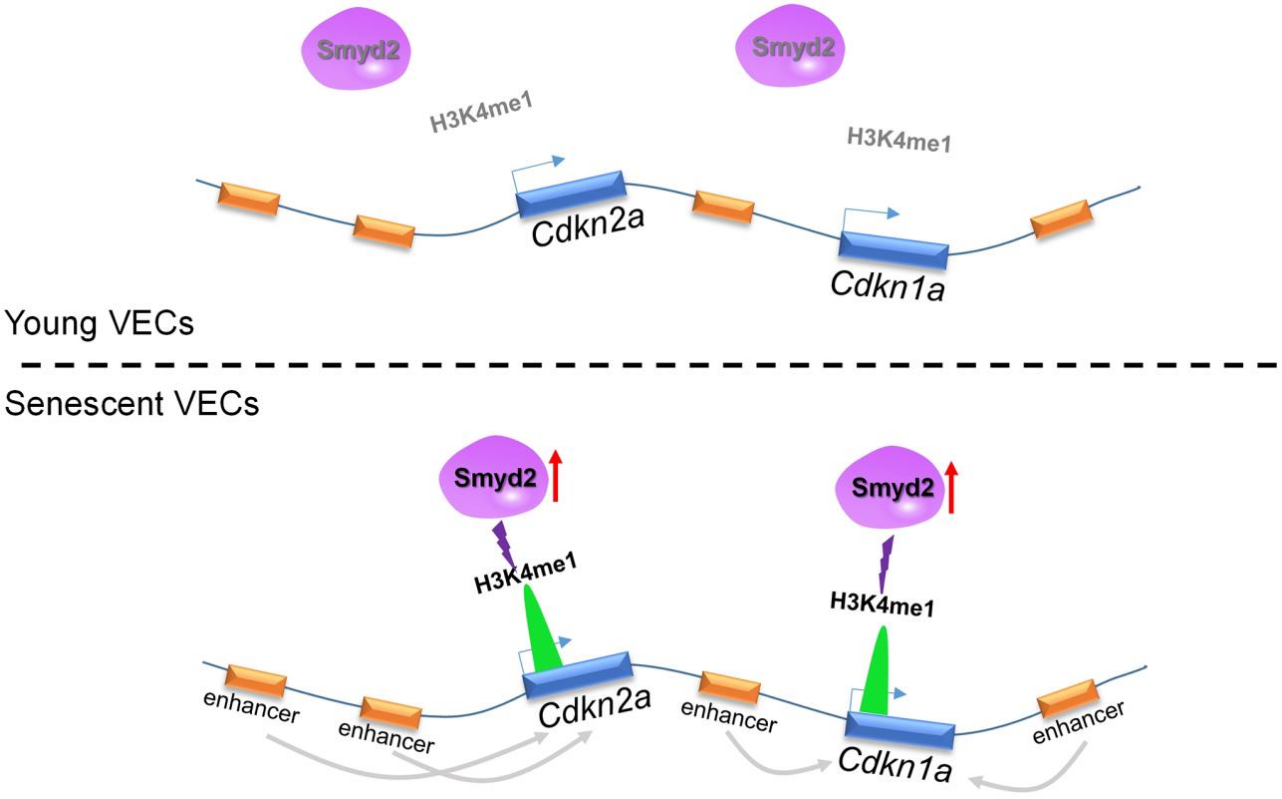
Ang II-induced Smyd2 upregulation activates enhancers adjacent to *Cdkn2a* (p16) and *Cdkn1a* (p21) genes by enhancing H3K4me1 enrichments and further promotes genes transcription, resulting in senescence-associated phenotypes and the development of vascular aging-related diseases.

In the present work, we identified Smyd2 as a vascular aging driver that may be a potential ’druggable’ target to ameliorate vascular aging-related diseases. Here we found that Smyd2 was upregulated in both vascular aging mice and VECs senescence model. Smyd2 deficiency attenuated while Smyd2 overexpression promoted senescence associated phenotypes. In addition, Smyd2 knockdown or heterozygous knockout mice ameliorate senescence-associated phenotypes upon Ang II infusion *in vivo*. Further mechanistic investigations revealed that Ang II-induced Smyd2 upregulation activates enhancers adjacent to *Cdkn2a* (p16) and *Cdkn1a* (p21) genes by enhancing H3K4me1 enrichments and further promoted genes transcription, resulting in senescence-associated phenotypes (Figure 8). Collectively, our findings reveal an epigenetics-based mechanism that drives specific histone modification status alterations by Smyd2 during vascular aging-associated diseases, shedding light on a potential therapeutic trigger in vascular aging.

It is noteworthy that Ang II is reported to be a pro-inflammatory and pro-atherogenic growth factor and contributes to the pathogenesis of cardiovascular diseases (CVDs) [31]. In this regard, Ang II is chosen as an ideal stimulant to induce vascular senescence. Furthermore, p21 and p16 are cyclin-dependent kinase inhibitors (CDKis) encoded in the *Cdkn1a* (p21^CIP^) and *Cdkn2a* (p16^INK4a^) loci, respectively, and activation of p21 and/or p16 drive cell cycle arrest and further result in cellular senescence [32, 33]. In addition, SA-β-Gal staining was performed to detect the beta-galactosidase activity in senescence cells, which is widely used as the biomarker of senescence [34]. Besides, EdU incorporation was also used to detect the DNA replication activity to verify the senescence state [35]. Therefore, consistent with our previous work [23], we here demonstrate that Ang II could induce senescence-related phenotypes both *in vitro* and *in vivo*, as indicated that increased SA-β-Gal activity, decreased EdU incorporation, upregulated senescence markers (p53, p21 or p16) and the proinflammatory mediators (iNOS, VCAM-1, COX-2 or IL-6).

It is also well documented that the vascular endothelium is a single layer of cells adjacent to the lumen of blood vessels and plays an important physiological role in vascular homeostasis including maintenance of blood fluidity, modulation of pro-inflammatory molecule production, and neovascularization. The VECs senescence induces vascular structural and functional changes and contributes to the progression of cardiovascular diseases in aging [36]. In light of this, we found Smyd2 knockdown or heterozygous knockout mice ameliorates endothelial inflammation (iNOS, VCAM-1 and COX-2) upon Ang II infusion *in vivo*, which revealed that targeting Smyd2 possibly unveiled a novel therapeutic candidate of age-related endothelial inflammation.

Subsequent studies have revealed that epigenetic changes are closely related to cardiovascular aging, such as DNA methylation and post-translational modification of histones [10]. Among them, histone methylations are mediated by different enzymes that transfer one, two, or three methyl groups to the lysine, then alter the chromatin architecture and influence the genome stability and lifespan [37]. However, the mechanistic role of histone methylation in vascular aging has yet to be elucidated. Smyd2 is a methyltransferase that methylates both the histone on lysine 36 and 4 (H3K36 and H3K4) and non-histone proteins such as p53 and RB1 [38]. Importantly, we have previously shown that Smyd3 drives vascular senescence by transcriptionally promoting p21 expression [23] and meanwhile, Smyd3 and Smyd2 have about 30% homology in the SET and MYND domains [39]. Thus, we are curious whether Smyd2 also has an impact on vascular senescence. Just as we expected, Smyd2 increased in both Ang II-induced vascular aging mice and VECs senescence model, implicating Smyd2 was associated with vascular endothelial senescence. Furthermore, genetic or pharmacologic suppression of Smyd2 collapses the expressions of senescence markers and proinflammatory mediators *in vitro*. Meanwhile *in vivo*, both Smyd2 knockdown by sh*Smyd2* and *Smyd2*^+/−^ mice ameliorate vascular aging phenotypes upon Ang II infusion. The above data strongly support that Smyd2 may be a novel epigenetic driver of vascular aging.

As known, the active distal states of the open chromatin could be labeled as enhancers which are characterized by the abundance of H3K4me1 and H3K27ac modification. Recently it has been reported that senescence-activated enhancers can positively regulate robust changes in SASP expressions during MEFs replicative senescence, accelerating the senescence progression [21]. Here in this study, we performed ChIP-seq using H3K4me1, H3K27ac, and H3K4me3 antibodies to profile the global enhancer activities and investigate the molecular evidence of altered enhancers in Ang II-induced senescent endothelial cells. As the track figures showed, remote enhancer elements engaged by Ang II were observed where located approximately 10 kb distal to *Cdkn2a*(p16) and *Cdkn1a* (p21) genes. These remote enhancer elements were at an active enhancer state which is characterized by high H3K27ac and H3K4me1 levels. Furthermore, Smyd2 drove a hyper-methylated chromatin state via H3K4me1 and activated the enhancer elements adjacent to *Cdkn1a* and *Cdkn2a* genes, and further induced the senescence-related phenotypes.

Targeting Smyd2 is possibly unveiling a novel therapeutic agent for the treatment of age-related vascular dysfunction. It is also noteworthy that Smyd2-mediated endothelial cell senescence exits in not only cellular senescence (Ang II-induced RAECs senescence and the replicative RAECs senescence) and animal vascular aging models (Ang II-induced vascular aging mice, 2K1C mice and db/db mice), but also human vascular disease tissues (human atherosclerotic samples), indicating it is an evolutionarily conserved mechanism.

Taken together, we profile the Ang II-regulated enhancers repertoires in RAECs and show that Smyd2-activated enhancers adjacent to *Cdkn2a* (p16) and *Cdkn1a* (p21) genes by enhancing H3K4me1 enrichments and drove Ang II-induced VECs senescence, implying that the co-operation between epigenetics and enhancer dynamics perform a key mechanism driving aging-related diseases. Our data establish a repertoire of Smyd2 in vascular aging and uncover that Smyd2 may be an attractive candidate for anti-aging therapy.

## METHODS

### Reagents

Reagents and antibodies used in this study were obtained as follows: angiotension II (Ang II) was purchased from Meilunbio (Meilun Biotechnology, Dalian, China); the Smyd2 specific inhibitor LLY-507 was purchased from MedChem Express (MCE, USA); Smyd2, p53, p21, p16 and γ-H2AX antibodies were obtained from Cell Signaling Biotechnology (Danvers, MA, USA); H3K4me3, H3K4me1 and H3K27ac antibodies were purchased from Abcam (Cambridge, MA, USA); COX-2, VCAM-1 and GAPDH were purchased from Proteintech (Rosemont, IL, USA); p-ATM and p-Chk2 antibodies were purchased from Absci (MD, USA); IL-6 and iNOS antibodies were purchased from ABclonal (Wuhan, China).

### Animal studies

All mice were housed and bred in pathogen-free conditions with a 12 hours light/dark cycle at 22 to 24°C and access to food and water ad libitum. 8–12 weeks old male C57BL/6J or *Smyd2*^+/−^ mice were used to establish vascular senescence model in our study.

To establish the vascular aging mice model, Ang II (1.5 mg/kg per day) was delivered by osmotic minipump (Model 2004, ALZA Scientific Products, Mountain View, CA, USA) in mice for 4 weeks. Meanwhile, the lentiviral vector expressing Smyd2 shRNA (sh*Smyd2*) was constructed to induce the knockdown of Smyd2 *in vivo* and the concentrated viral suspension (150 μl) was intravenously injected via the tail vein every 7 days.

The Smyd2 heterozygous (*Smyd2*^+/−^) mice was constructed as reported previously [15]. Then male *Smyd2*^+/−^ mice and the wild-type littermates (WT) were infused with saline or Ang II for 4 weeks.

The diverse vascular aging-related diseases mice model were established as follows: The two-kidney, one-clip (2K1C) hypertension mice model was established as previously reported [23]. Briefly, 6–8 weeks C57BL/6J male mice were anesthetized with isoflurane and exposed the left kidney. The left renal artery was isolated and placed a U-shaped sterile stainless-steel clip (0.12 mm internal diameter) around. Sham group underwent the same procedure without the renal artery clip insertion. Moreover, the db/db mice and its control db/m mice were obtained from Gempharmatech Co., Ltd. (China). Then western diet was used to feed the ApoE^−/−^ mice for 12 weeks to establish the classical atherosclerosis mouse model. These above model mice were sacrificed and harvested the arteries for further examination.

### Plasma construction, lentivirus generation and infection

pLKO.1-shRNA encoding shRNAs targeting Smyd2 was conducted using the shRNA-expressing lentiviral vectors, which were purchased from Sigma-Aldrich. Full length of Smyd2 cDNA were constructed by inserting PCR amplified fragments into pCDH-EF1-MSC-T2A-Puro lentiviral vector (System Biosciences, CA). The knockdown or overexpressing plasmids were verified by DNA sequencing.

Lentiviral particles were obtained by transfecting HEK293T cells with knockdown or overexpressing plasmids and packaging vector PMD2.G and psPAX2. The lentiviral particles were harvested from the supernatant of HEK293T cells at 48 h and 72 h, respectively and then were used to transduce RAECs after filtration with 0.45 μm filters.

### Cell culture and induction

Rat aorta endothelial cells (RAECs) were primary isolated from thoracic aortas of male Sprague-Dawley rats as we previously reported [23]. Briefly, thoracic aortas were isolated and make the intima outward to be digested with type II collagenase. Then the intima of the vessel was attached to the flask and cells were slowly crawl out. Cells with CD31 antibody positive staining but α-SMA antibody negative staining and the classical ‘hill and valley’ morphology were identified as RAECs for the follow-up studies.

Human Umbilical Vein Endothelial Cells (HUVECs) used in this study were cultured in DMEM (Gibco) supplemented with 10% FBS (Gibco) and 1% penicillin/streptomycin in a humidified atmosphere containing 5% CO_2_ at 37°C.

Ang II (100 nM) was added to RAECs or HUVECs for 48 h to establish the *in vitro* senescence model. LLY-507 (3 μM) or Smyd2 siRNA (15 nM) was added to incubate with cells for 4 h or 24 h, respectively before Ang II treatment.

### Senescence-associated β-galactosidase (SA-β-gal) staining

The SA-β-gal activity was monitored using the Senescence Cells Histochemical Staining Kit (Sigma, CS0030, USA) according to the manufacturer’s instructions. Briefly, RAECs were washed three times with PBS and fixed with 1% formaldehyde solutions for 7 min. then RAECs were incubated overnight with the staining mixture at 37°C overnight. The images were acquired by ZESS light microscope.

### The proliferation analysis

The proliferation analysis of Ang II-induced RAECs was detected using Ethynyl-2’-deoxyuridine (EdU) Labeling and Detection Kit (KeyGEN BioTECH, Jiangsu, China) to measure the EdU incorporation into cellular DNA according to the manufacturer's instructions. The nuclei were stained with DAPI and the EdU positive cells images were acquired by ZESS inverted fluorescent microscope.

### Western blotting

The artery tissues were lysed with RIPA buffer (Pierce, Rockford, IL, USA) while RAECs were lysed with LDS Sample Buffer (Thermo, Invitrogen, CA, USA) containing 1% protease and phosphatase inhibitor cocktail (Sigma, St Louis, USA) and 5% β-mercaptoethanol. Whole protein lysates samples were separated by SDS-PAGE and blotted to nitrocellulose (NC) membrane. The protein bands were incubated with corresponding primary antibodies at 4°C overnight and then were incubated with anti-rabbit and anti-mouse IgG HRP-conjugated secondary antibodies the next day. Chemiluminescence was generated and detected by ChemiDoc^+^ (Bio-RAD, Hercules, CA, USA).

### Immunohistochemistry (IHC) staining

The expression of Smyd2 protein in the vessel endothelium of Ang II-infusion mice was determined by IHC stained with Smyd2 antibody according to standard procedures. Briefly, paraffin-embedded arterial sections were incubated with anti-Smyd2 (21290-1-AP, Proteintech) at 4°C overnight. The second day, an appropriate secondary antibody was used for 1.5 h, then followed by visualization with 3, 3-diaminobenidine (DAB). Counterstaining with hematoxylin for 10 s, then wash with distilled water for 10 min. The images were captured by using a fluorescence microscope (Axio Scope.A1, Carl Zeiss Imaging Systems).

### Immunofluorescence staining

RAECs were seeded on microscope coverslips placed in 24-well plates. RAECs or the arterial sections were fixed with 4% paraformaldehyde for 15 min, washed three times for 5 min with PBS, followed by permeabilization with 0.25% Triton X-100 in PBS 10 min. Next, the slides were blocked in PBS with 5% goat serum for 30 min and incubated overnight with primary antibodies at 4°C. Appropriate secondary antibodies were added and incubated with slides for 1.5 h at room temperature. The nuclei were counterstained with DAPI. Images were captured by using a fluorescence microscope (Axio Scope.A1, Carl Zeiss Imaging Systems).

### Small interfering RNA (siRNA) transfection

For Smyd2 gene knockdown, 30% to 50% confluence of RAECs were transfected with Smyd2 siRNA (si Smyd2) and scrambled siRNA (si CTL), which were produced by GenePharma (Shanghai, China). Smyd2 siRNA (15 nM) mixed with Lipofectamine RNAiMax (Invitrogen) in Opti-MEM (Gibco) and incubated for 5 min at room temperature. Then the complexes were added to RAECs and incubated for 24 h. Western blot or RT-qPCR assays were performed at 72 h or 48 h after transfection.

### RT-qPCR

Total RNA of RAECs or vascular tissue were extracted using RNAiso Plus (TaKaRa Biotechnology, Dalian, China) and then reverse-transcribed into cDNA using the PrimeScript 1st Strand cDNA Synthesis Kit (Takara) according to the manufacturer’s directions. Gene expressions of senescence markers and proinflammatory mediators were quantified using iCycler iQ system (Bio-Rad, Hercules, CA, USA). The primer sequences were listed in Supplementary Table 1.

### ChIP-seq library construction and analysis

ChIP-seq libraries were constructed according to the protocol established previously [23], and H3K4me1 (8895, CST), H3K27ac (ab4729, abcam) and H3K4me3 (ab8580, abcam) antibody were used during the immunoprecipitation step. After quality inspection, ChIP-seq libraries were sequenced by Illumina HiSeq platform, and paired-end reads of 150 nt length were obtained. The quality of the raw reads (of 150 nt length) was first evaluated through FastQC (http://www.bioinformatics.babraham.ac.uk/projects/fastqc), and then the 3’ end 60nt were trimmed off to remove the nucleotide of low sequencing quality and adaptor sequence originated from the running off of relatively short inserted fragments.

The ChIP-seq data was analyzed as previously described (Yang et al., 2020). Bdgdiff (a sub-tool of MACS2) was used to find the significant different H3K4me1, H3K4me3, H3K27ac distribution between the control and Ang II-treated RAECs. Regions of at least 1 kb-distant from TSS or H3K4me3 peaks were defined as the enhancer region and used to search for the potential binding site of Smyd2.

### ChIP-PCR

Smyd2 antibody (9734, CST) were used during the immunoprecipitation step before PCR. Taq DNA Polymerase (Yeasen) was used for the PCR step, and PCR primers for selected peaks were listed in Supplementary Table 2. Thermal cycling was carried out as follows: 94°C for 30 s; 30 ~ 40 cycles of 94°C for 30 s, 52~58°C for 30 s and 72°C for 30 s; 72°C for 10 min; hold at 4°C. PCR product was visualized in a 2% agarose gel stained by EB dye.

### Statistical analysis

GraphPad Prism 7.0 was used to analyze the data which were expressed as mean ± SEM. Differences of means were analyzed by using one-way ANOVA to compare one variable in multiple groups or two-way ANOVA with the Bonferroni’s *post hoc* test for multiple groups, and when comparing between two groups using unpaired Students *t*-test. *p* < 0.05 was regarded as statistically significant.

## Supplementary Materials

Supplementary Figures

Supplementary Tables
