# Supplementary material for: Histone methyltransferase Smyd2 drives vascular aging by its enhancer-dependent activity
Source: Aging (Albany NY). 2022 Dec 28;15(1):70–91. doi: 10.18632/aging.204449 (PMC9876634; doi:10.18632/aging.204449)
Supplement: Supplementary Figures [file aging-15-204449-s001.pdf]

SUPPLEMENTARY FIGURES

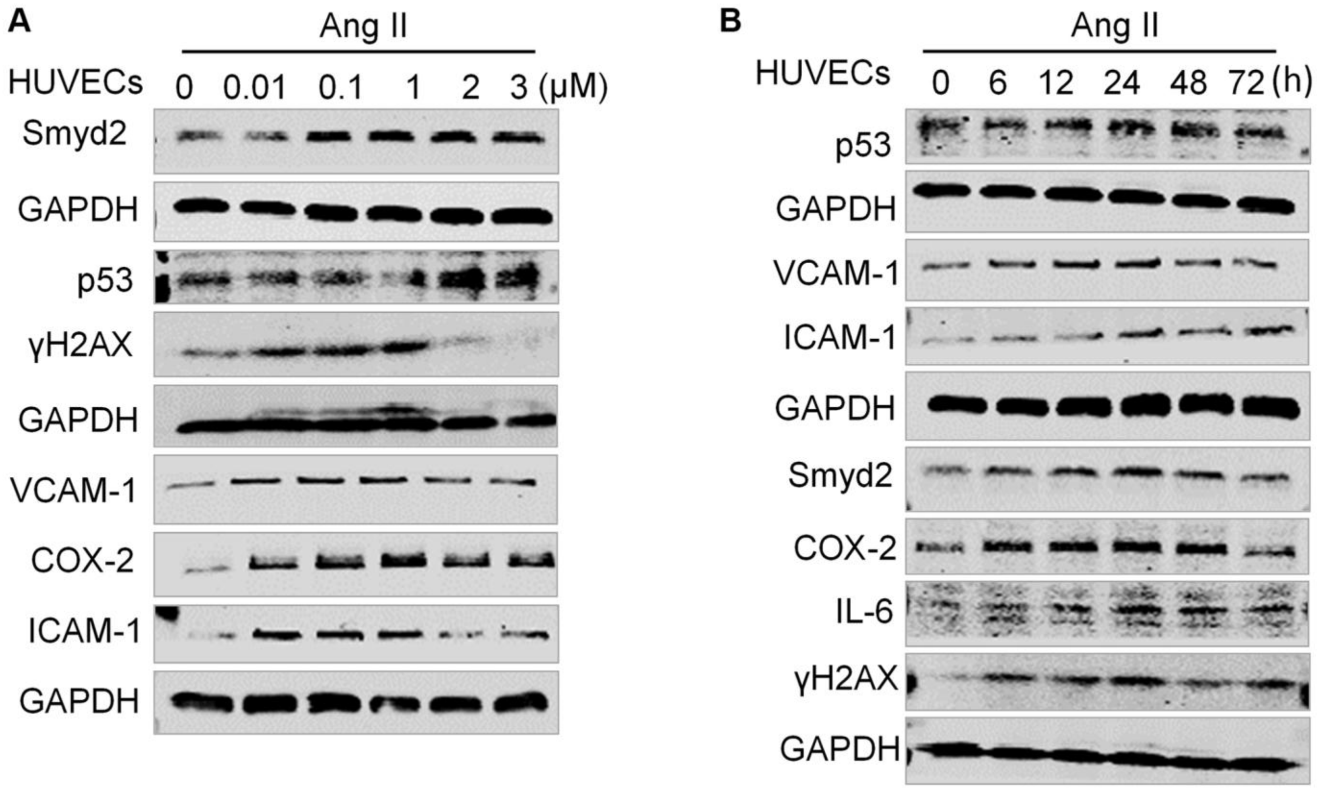

Supplementary Figure 1. Ang II led to Smyd2 upregulation in both time- and dose-dependent manner in HUVECs. (**A**) Smyd2 and the senescence related phenotype markers were increased in a dose-dependent manner in Ang II-induced HUVECs. (**B**) The protein expressions of Smyd2 and the senescence related phenotype markers were increased in a time-dependent manner in Ang II-induced HUVECs.

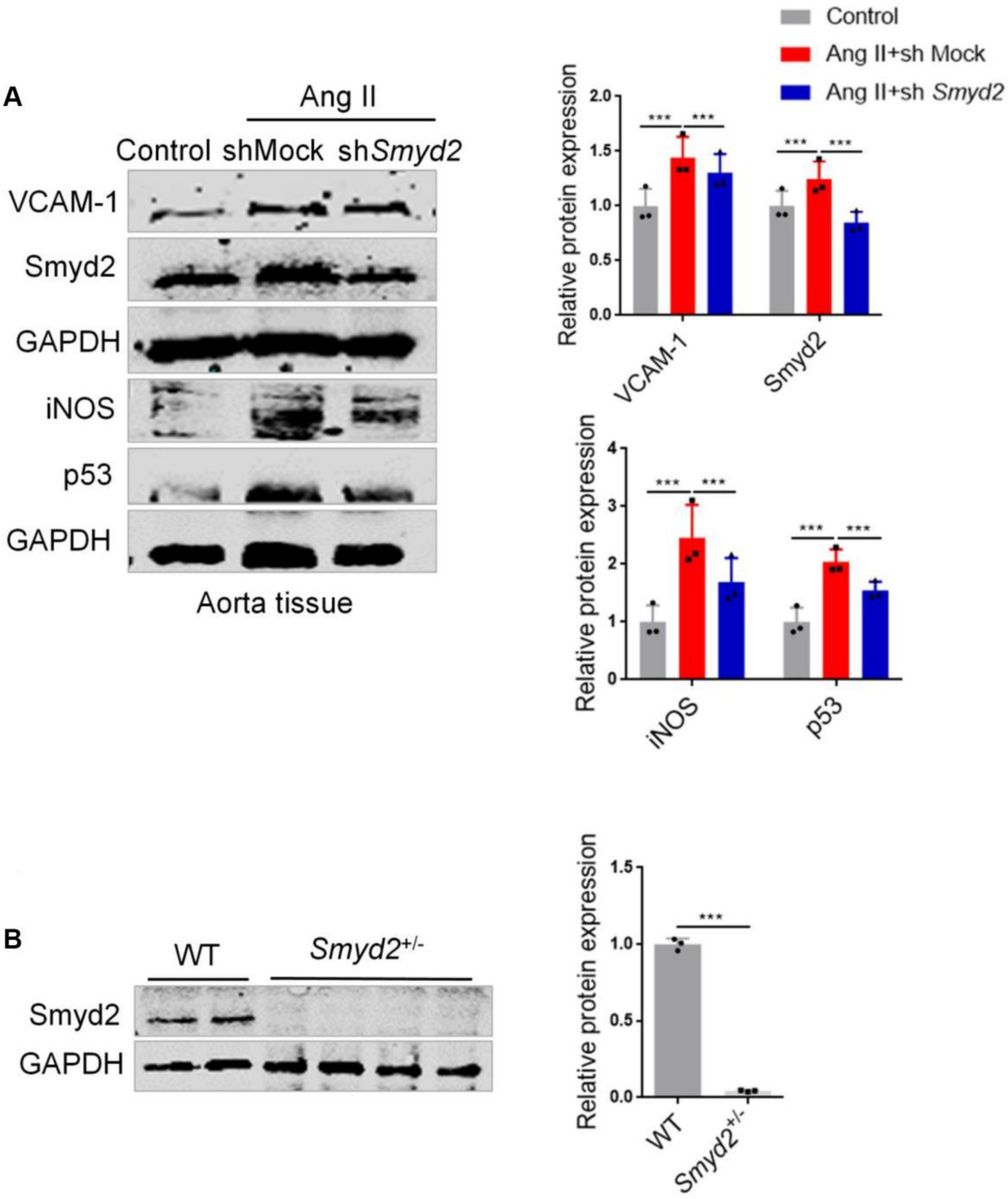

Supplementary Figure 2. Smyd2 knockdown ameliorates senescence-associated phenotypes upon Ang II infusion. (**A**) Lentivirus-mediated knockdown of Smyd2 (sh*Smyd2*) were performed in Ang II-infused mice. Smyd2, the senescence marker (p53) and the pro-inflammatory molecules (iNOS and VCAM-1) expressions were detected by immunoblots. (**B**) The verification of Smyd2 protein expression in the aorta of *Smyd2*$^{+/-}$ mice was performed by western blots. Data are presented as the mean ± SEMs, $n = 3$. $^{***}p < 0.001$.

	Num of peaks	At least 1 kb-distant from TSS or H3K4me3 peaks		
		Filtered peaks	Up	Down
H3K27ac	11,202	7,580	2,256	125
H3K4me1	48,332	41,649	8,143	3,277
H3K4me3	13,171			
IgG	207			

Supplementary Figure 3. The analysis of ChIP-seq data for identifying active enhancers. ChIP-seq analysis was performed following lysis of cross-linked RAECs upon Ang II treatment, using antibodies recognizing H3K27ac for active enhancers, H3K4me1 for candidate enhancers and H3K4me3 for active promoter regions to profile the global enhancer activities and investigate the molecular evidence of altered enhancers in Ang II-triggered RAECs. MACS2 was used to call peaks significantly enriched with ChIP-seq reads, using the *Q* value (corrected *p* value) < 0.001 as the cutoff. To eliminate the disturbance from transcription starting site (TSS) or promoters, H3K4me1 and H3K27ac peaks should be at least 1 kb from a known TSS site or H3K4me3 peaks. Here we chose the peaks with changes of normalized coverage greater than 2-fold as changed peaks corresponding to Ang II treatment.

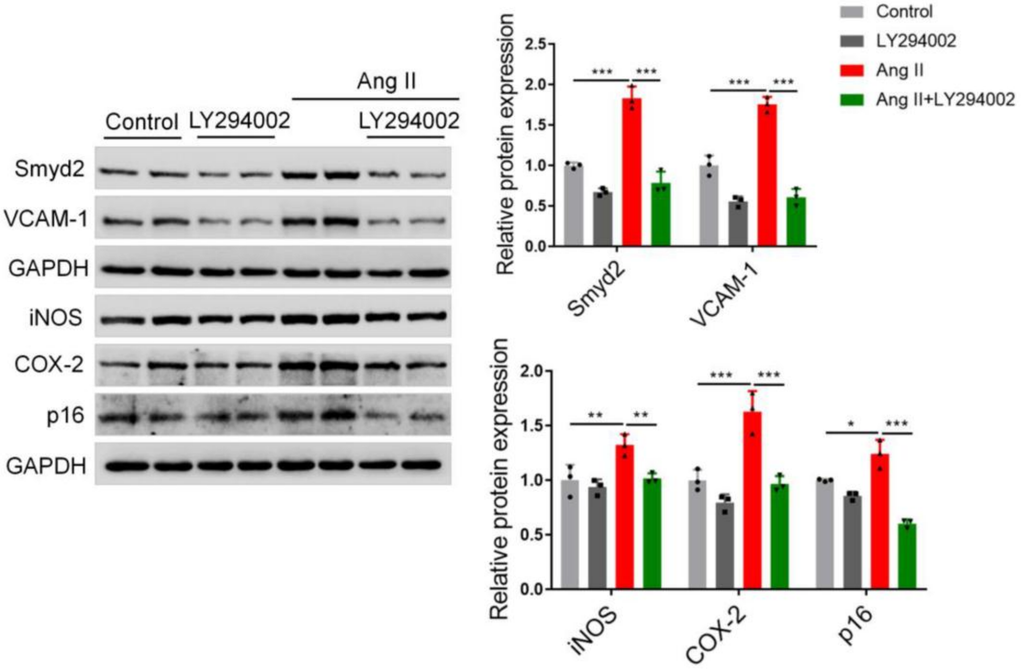

Supplementary Figure 4. Ang II-induced Smyd2 upregulation is mediated by the PI3K/Akt pathway. The protein levels of Smyd2 and the vascular senescence related phenotypes (VCAM-1, iNOS, COX-2 and p16) were detected by immunoblots in Ang II-induced RAECs pretreated with the PI3K/Akt signaling pathway inhibitor LY294002. The statistical analysis of relative protein expressions is shown in the figure right. Data are presented as the mean ± SEMs, $n = 3$. $^{*}p < 0.05$, $^{**}p < 0.01$, $^{***}p < 0.001$.
