# Supplementary material for: Histone methyltransferase Smyd2 drives vascular aging by its enhancer-dependent activity
Source: Aging (Albany NY). 2022 Dec 28;15(1):70–91. doi: 10.18632/aging.204449 (PMC9876634; doi:10.18632/aging.204449)
Supplement: Supplementary Tables [file aging-15-204449-s002.pdf]

SUPPLEMENTARY TABLES

Supplementary Table 1. The PCR primers for RT-qPCR.

Name	Sequence
Rat_*Smyd2*_F	TCCCCTAGTGAGCTGTTGGA
Rat_*Smyd2*_R	GGCCCATGTACAGTCTTCCC
Rat_*Cdkn1a*_F	GTGGACAGTGAGCAGTTGAG
Rat_*Cdkn1a*_R	TCAGGTAGATCTTGGGCAGC
Rat_*Il6*_F	CTCTCCGCAAGAGACTTCCA
Rat_*Il6*_R	CTCCTCTCCGGACTTGTGAA
Rat_*Nos2*_F	CAGCCTGTGAGACGTTCGAT
Rat_*Nos2*_R	CCCATGTTGCGTTGGAAGTG

Supplementary Table 2. The PCR primers for ChIP-PCR.

Name	Sequence
Peak 1-F	GTACTTGAGCCCGGTTGCTT
Peak 1-R	TAAAGGCACTACGAACCCGC
Peak 2-F	TTTCCCCGCTTTTTGTGCTG
Peak 2-R	TGTTGGCGGTGTTATCCAGT
Peak 3-F	GGTAGCTTTGGTGGGAGTCG
Peak 3-R	CTCCAAGGTCACCAGAACGG
Peak 4-F	AGGCTGATCTGAGGCATACG
Peak 4-R	GTTGAGAGGGCAATGACCGT
